# Internet addiction and residual depressive symptoms among clinically stable adolescents with major psychiatric disorders during the COVID-19 pandemic: a network analysis perspective

**DOI:** 10.1038/s41398-023-02468-5

**Published:** 2023-06-03

**Authors:** Hong Cai, Yan-Jie Zhao, Fan He, Shu-Ying Li, Zong-Lei Li, Wu-Yang Zhang, Yao Zhang, Teris Cheung, Chee H. Ng, Sha Sha, Yu-Tao Xiang

**Affiliations:** 1 Unit of Psychiatry, Department of Public Health and Medicinal Administration, & Institute of Translational Medicine, Faculty of Health Sciences, University of Macau, Macao SAR, China; 2 Centre for Cognitive and Brain Sciences, University of Macau, Macao SAR, China; 3 Institute of Advanced Studies in Humanities and Social Sciences, University of Macau, Macao SAR, China; 4 The National Clinical Research Center for Mental Disorders & Beijing Key Laboratory of Mental Disorders Beijing Anding Hospital & the Advanced Innovation Center for Human Brain Protection, Capital Medical University, School of Mental Health, Beijing, China; 5 Department of Psychiatry, The First Affiliated Hospital of Zhengzhou University, Zhengzhou, China; 6 Department of Psychiatry, Xiamen Xianyue Hospital, Xiamen, China; 7 Department of Pediatric Development and Behavior, The third Affiliated Hospital of Zhengzhou University, Zhengzhou, Henan province China; 8 Huashan Hospital, Fudan University, Shanghai, China; 9 School of Nursing, Hong Kong Polytechnic University, Hong Kong SAR, China; 10 Department of Psychiatry, The Melbourne Clinic and St Vincent’s Hospital, University of Melbourne, Richmond, VIC Australia

**Keywords:** Psychiatric disorders, Addiction

## Abstract

To assess the inter-relationships between residual depressive symptoms (RDS) and Internet addiction (IA) using network analysis among clinically stable adolescents with major psychiatric disorders during the COVID-19 pandemic. RDS and IA were assessed using the Patient Health Questionnaire-9 (PHQ-9) and the Internet Addiction Test (IAT), respectively. Central symptoms and bridge symptoms in the network model were examined. A total of 1,454 adolescents met the study criteria and were included in the analyses. The prevalence of IA was 31.2% (95% CI: 28.8%-33.6%). In the network analysis, the nodes IAT15 (“Preoccupation with the Internet”), PHQ2 (“Sad mood”), and PHQ1 (“Anhedonia”) were the most central symptoms in the IA-RDS network model. Bridge symptoms included IAT10 (“Sooth disturbing about your Internet use”), PHQ9 (“Suicide ideation”), and IAT3 (“Prefer the excitement online to the time with others”). Additionally, PHQ2 (“Sad mood”) was the main node linking “Anhedonia” to other IA clusters. Internet addiction was common among clinically stable adolescents with major psychiatric disorders during the COVID-19 pandemic. Core and bridge symptoms identified in this study could be prioritized as targets for the prevention and treatment of IA in this population.

## Introduction

Coronavirus disease 2019 (COVID-19) caused by Severe Acute Respiratory Syndrome Coronavirus 2 (SARS-CoV-2) was declared a pandemic on March 11, 2020, by the World Health Organization (WHO) [1]. The COVID-19 pandemic and relevant public health measures such as lockdown, social distancing, masks wearing, frequent hand hygiene, restriction of recreational activities, and school closure were associated with an increased risk of mental health problems in many vulnerable populations. Of note, children and adolescents had an increased risk of depression, anxiety and post-traumatic stress disorder (PTSD), and Internet addiction (IA) [2–6].

Internet addiction, a growing public health concern, is common among adolescents. A recent meta-analysis found that the overall prevalence of IA in adolescents was 13.62% [7]. Of note, the prevalence of IA had increased during the COVID-19 pandemic. A recent study found that the prevalence of self-reported IA and problematic internet use was 33.37% among children and adolescents aged 6 to 18 years old in China [8]. Another study reported that the prevalence of Internet abuse behavior was 34.7% among Italian adolescents during the COVID-19 pandemic [9], with factors such as gender, age, depression severity, impulsivity, co-dependency emotion, duration of Internet use, and stress as correlates [8, 10, 11]. Compared to the pre-pandemic period, the frequency and average duration of Internet use among adolescents increased during the COVID-19 pandemic [8, 10, 12], which could increase the risk of IA. Research has identified that the relationships between addiction behaviors (e.g., Internet addiction) and psychiatric symptoms are bidirectional [13, 14] according to the cognitive-behavioral model [15, 16]. Psychiatric symptoms/disorders could trigger addictive behaviors [17, 18], while addictive behaviors could also increase the risk of psychiatric symptoms/disorders [19]. Additionally, addiction behaviors (e.g., Internet addiction) often co-exist with psychiatric symptoms [14], including residual depressive symptoms (RDS) that are defined as persisting symptoms following partial treatment response or remission in patients with the psychiatric disorder [20], which may lead to more severe health outcomes compared to having IA alone [14]. RDS are common in patients with major psychiatric disorders such as major depressive disorders (MDD) [21], bipolar disorders (BD) [22], and attention deficit hyperactivity disorder (ADHD) [23]. Although psychosocial interventions and pharmacological treatments may have effective response rates, only a small proportion of patients with major psychiatric disorders achieve complete remission of symptoms [24–27]. There are underlying biological mechanisms shared by both addictive behaviors and psychiatric disorders, which involve the 5HTTLRP gene [28, 29], certain brain areas (e.g., altered activity in the anterior and posterior cingulate cortices and attenuated front-striatal top-down control [30]) and serotonin dysfunction [14]. However, most studies on RDS and IA were only based on syndromal assessment using standard scale total scores [31], even though both RDS and IA consist of various individual symptoms with possibly different psychoneurological mechanisms [32, 33].

To address these concerns, network analysis offers a novel approach to visualizing the interactions between various symptoms of psychiatric disorders/syndromes [32] and estimating the strength and nature of associations among symptoms [34, 35]. In Network analysis, can identify central (influential) symptoms have the most important role within a network model [36, 37] that help develop and maintain psychiatric disorders/syndromes. In network theory, nodes refer to psychiatric symptoms and edges between nodes reflect relationships between symptoms, while activation can spread from one symptom to other symptoms through the network in the model [33, 35]. Central (influential) nodes, can be potential therapeutic targets because their levels of activation may directly affect other intimately connected nodes that will also be (in)activated [32]. Targeting central symptoms in preventive measures and interventions may achieve more effective outcomes [32, 33]. In contrast, bridge nodes (symptoms) have the strongest links with comorbid disorders/syndromes [38], and can transfer symptom activation from one disorder/syndrome to another disorder/syndromes, which may be targeted for treatments intended to reduce or prevent comorbid problems. Certain network analysis studies on IA and related problems have been reported. For instance, a network analysis of IA among Japanese adolescents with autism spectrum disorder revealed that “Defensive and secretive behaviors” and “Concealment of Internet use” were the central symptoms in the network [39]. Another two network analysis showed that the core symptoms of students’ problematic smartphone use included “Loss of control” [40, 41]. However, to date, no network analysis studies have evaluated the inter-relationships between IA and RDS among adolescents with major psychiatric disorders.

Therefore, we examined the relationships between comorbid IA and RDS among clinically stable adolescents with major psychiatric disorders during the COVID-19 pandemic from a network analysis perspective. Given the negative consequence of the COVID-19 pandemic on mental health, such as the increased risk of IA in adolescents [8, 10, 12], we hypothesized that IA would be common among clinically stable adolescents with psychiatric disorders.

## Methods

### Participants and procedure

This was a multicenter, cross-sectional survey carried out between April 29 and June 9, 2020, in three major tertiary mental health centers on children and adolescents located in northern (Beijing), southern (Fujian province), and central areas of China (Henan province) [42]. With the strict COVID strategy in China, there were only a very limited number of infected cases in China during the study period [43]. To be eligible, all participants were: (1) aged between 10 and 17 years; (2) outpatients receiving maintenance treatment for a major psychiatric disorder as defined by local health authorities of the participating hospitals (e.g., major depressive disorders (MDD), bipolar disorders (BD), and attention deficit hyperactivity disorder (ADHD), autism, tics, and psychosis and others); (3) clinically stable as judged by treating psychiatrists. Following previous studies [44, 45], those with changes in doses of psychotropic medications of less than 50% in the past three months were considered “clinically stable patients”; and (4) all participants provided verbal consent, and their guardians provided written informed consent. Participants’ primary psychiatric disorders according to the International Statistical Classification of Diseases and Related Health Problems, 10th Revision (ICD-10) [46] were recorded. This study was centrally approved by the ethics committee of Beijing Anding Hospital and other participating hospitals (No.: 202024XG-1).

### Measurement

Internet addiction was assessed using the validated Chinese version of the Internet Addiction Test (IAT) [47, 48]. The IAT comprises 20 items, with each rated from 1 (“rarely”) to 5 (“always”) with IAT total score of ≥50 being considered as having “Internet addiction” [49, 50] (Table S1). The IAT contains four domains, including “Reliance on online life”, “Relationships”, “Neglect of work”, and “Injurious effects on self-control”. The Chinese version of the IAT has been validated with acceptable psychometric properties (e.g., Cronbach’s alpha of α = 0.90) [47]. RDS was assessed using the validated Chinese version of the 9-item Patient Health Questionnaire (PHQ-9) [51, 52]. Each item score of the PHQ-9 ranges from 0 (“not at all”) to 3 (“almost every day”) (Table S2). The total score ranges from 0 to 27, with a higher score indicating more severe depressive symptoms [53–55]. The Chinese version of the PHQ-9 has been validated with satisfactory psychometric properties (e.g., Cronbach’s alpha of α = 0.85) [54].

### Statistical analyses

The network model of IA and RDS was computed using the R software [56]. We computed the polychoric correlations between all the items to investigate the edges of the network, and also estimated the Graphical Gaussian Model (GGM), with the graphic least absolute shrinkage and selection operator (LASSO) and Extended Bayesian Information Criterion (EBIC) model using the R package *“graph”* [57].

The importance of each node in the network was examined by estimating centrality indices of the network structure, with the R package *“graph”* [58]. Specifically, the centrality index of expected influence (EI) was computed for each node in the network (i.e., the sum of the weights of the connections, in absolute value), because EI is the most stable and interpretable centrality index [57]. Following a previous study [59], to reduce the number of spurious edges and improve the interpretability of results, network models were regularized using LASSO, a well-established algorithm for regularization that eliminates weak associations by removing potentially “false positive” edges from the models [57]. In addition, to examine nodes that more often fall on the shortest predictive pathways from Anhedonia (PHQ1) to other nodes, we computed node-specific predictive betweenness as a centrality measure. As betweenness is generally not a stable centrality measure [57], we used both nonparametric and case-drop bootstraps to investigate the degree of variability in-betweenness [57]. Node-specific predictive betweenness of anhedonia (i.e., how often a node lies on the pathways between two other nodes, always with the “Anhedonia” (PHQ1) node as either of them across 1000 nonparametric bootstrap iterations) was estimated [60–62].

Following previous studies [63, 64], the differences in network characteristics between male and female participants were compared using the R “*NetworkComparisonTest*” package (Version 2.2.1) [65] with 1000 permutations. The differences in network structure (e.g., distributions of edge weights), global strength (e.g., total absolute connectivity among the symptoms), and each specific edge between subsamples (i.e., females vs. males) were also examined. The researchers in each participating hospital checked the completion of the questionnaires prior to submission to minimize any missing values. Therefore, participants completed all the assessments.

## Results

A total of 1570 adolescent patients were invited to participate in this study, of whom 1454 met the study criteria and were included in the analyses. The prevalence of IA was 31.2% (95% CI: 28.8%–33.6%) among adolescent psychiatric outpatients during the COVID-19 pandemic. The mean score of RDS was 8.12 (Standard Deviation (SD):8.42) (Table 1). Mean, SDs, skewness, and kurtosis of all PHQ-9 and IAT items scores are presented in Supplementary Table 3.

**Table 1:** Socio-demographic characteristics of adolescents with major psychiatric disorders.

Variables	Total (*N* = 1454)	
	*N*	%
Male gender	564	38.8
Principal psychiatric disorder		
MDD	759	52.2%
BD	123	8.5%
ADHD	70	4.8%
Others	502	34.5%
	Mean	SD
PHQ-9 total	8.51	8.52
IAT	41.21	19.17

*MDD* major depressive disorder, *BD* bipolar disorder, *ADHD* attention deficit hyperactivity disorder, *PHQ-9* the 9-item Patient Health Questionnaire, *IAT* Internet Addiction test, *SD* standard deviation.

### Network structure

**Fig. 1:**
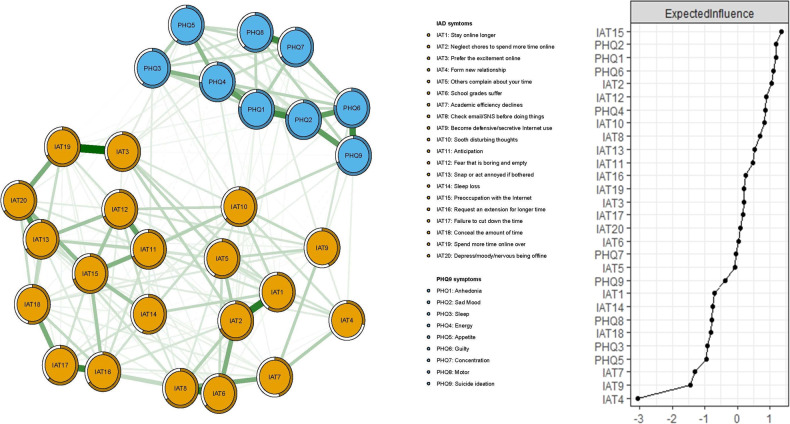
The network structure of Internet addiction and residual depressive symptoms in adolescents with psychiatric disorders in China during the COVID-19 pandemic.

Figure 1 presents the network structure of comorbid IA and RDS in adolescents with major psychiatric disorders. A total of 222 (54.7%) non-zero edges emerged out of 406 possible edges. The predictability of symptoms is shown as ring-shaped pie charts in Fig. 1, with a mean predictability of 0.64, indicating that, on average, 64% of each node’s variance could be accounted for by neighboring nodes in the model. The network model showed that the connection PHQ9 (“Suicide ideation”)—PHQ6 (“Guilty”) was the strongest positive edge in the RDS community, followed by PHQ8 (“Motor”)—PHQ7 (“Concentration”), and PHQ1 (“Anhedonia”)—PHQ2 (“Sad mood”). In the IA community, IAT3 (“Prefer the excitement online”)—IAT19 (“Spend more time online over”) were the strongest edge, followed by IAT8 (“Check email/SNS before doing things”)—IAT6 (“School grade suffer due to Internet use”), and IAT1 (“Stay online longer than intended”) - IAT2 (“Neglect chores to spend more time online”).

**Fig. 2:**
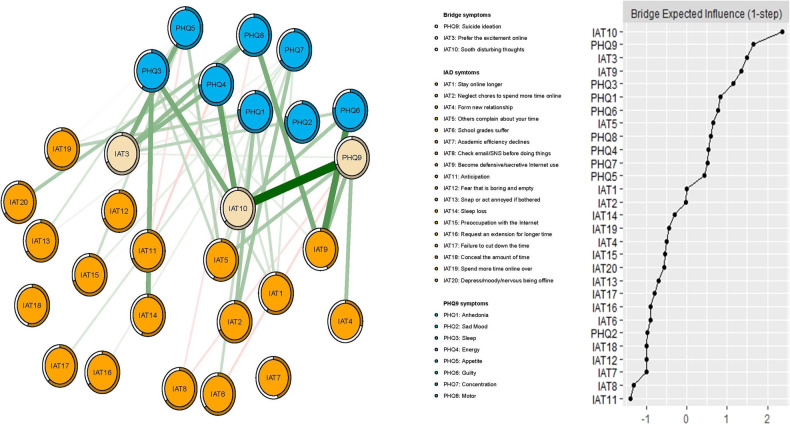
The network structure of Internet addiction and residual depressive symptoms in adolescents with psychiatric disorders in China during the COVID-19 pandemic only showed a bridge connection.

In terms of EI, the node IAT15 (“Preoccupation with the Internet”) had the highest EI centrality, followed by PHQ2 (“Sad mood”) and PHQ1 (“Anhedonia”) in the network (Fig. 1). In contrast, several other symptoms were marginal such as the IAT7 (“Check email/SNS before doing things you need to do”), IAT9 (“Become defensive/secretive about your Internet use”), and IAT4 (“Form a new relationship with online users”) (Fig. 1). In terms of bridge EI, IAT10 (“Sooth disturbing about your Internet use”) in the IA community was the most key bridge symptom linking the IA and RDS communities, followed by PHQ9 (“Suicide ideation”) and IAT3 (“Prefer the excitement online to the time with others”) (Fig. 2).

**Fig. 3:**
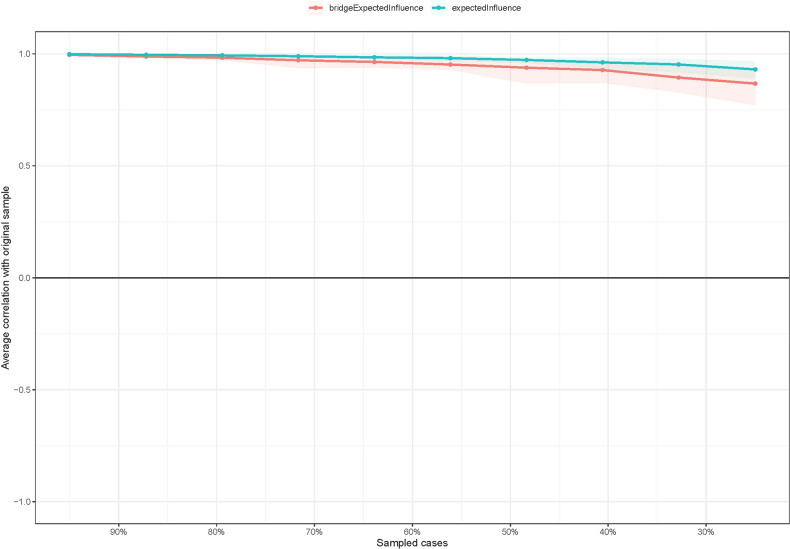
The stability of centrality and bridge centrality indices using case-dropping bootstrap.

For the network stability, the centrality of EI had an excellent level of stability (i.e., CS-coefficient = 0.75 (95% CI: 0.675-1)), which indicates that 75% of the sample could be dropped, and the structure of the network would not significantly change (Fig. 3). The results of the bootstrap 95% CI for edges and bootstrapped differences tests for edge weight are shown in Supplementary Fig. S1. The bootstrap difference test showed that most comparisons between edge weights were statistically significant (Supplementary Fig. S1).

### Node-specific predictive betweenness measure

**Fig. 4:**
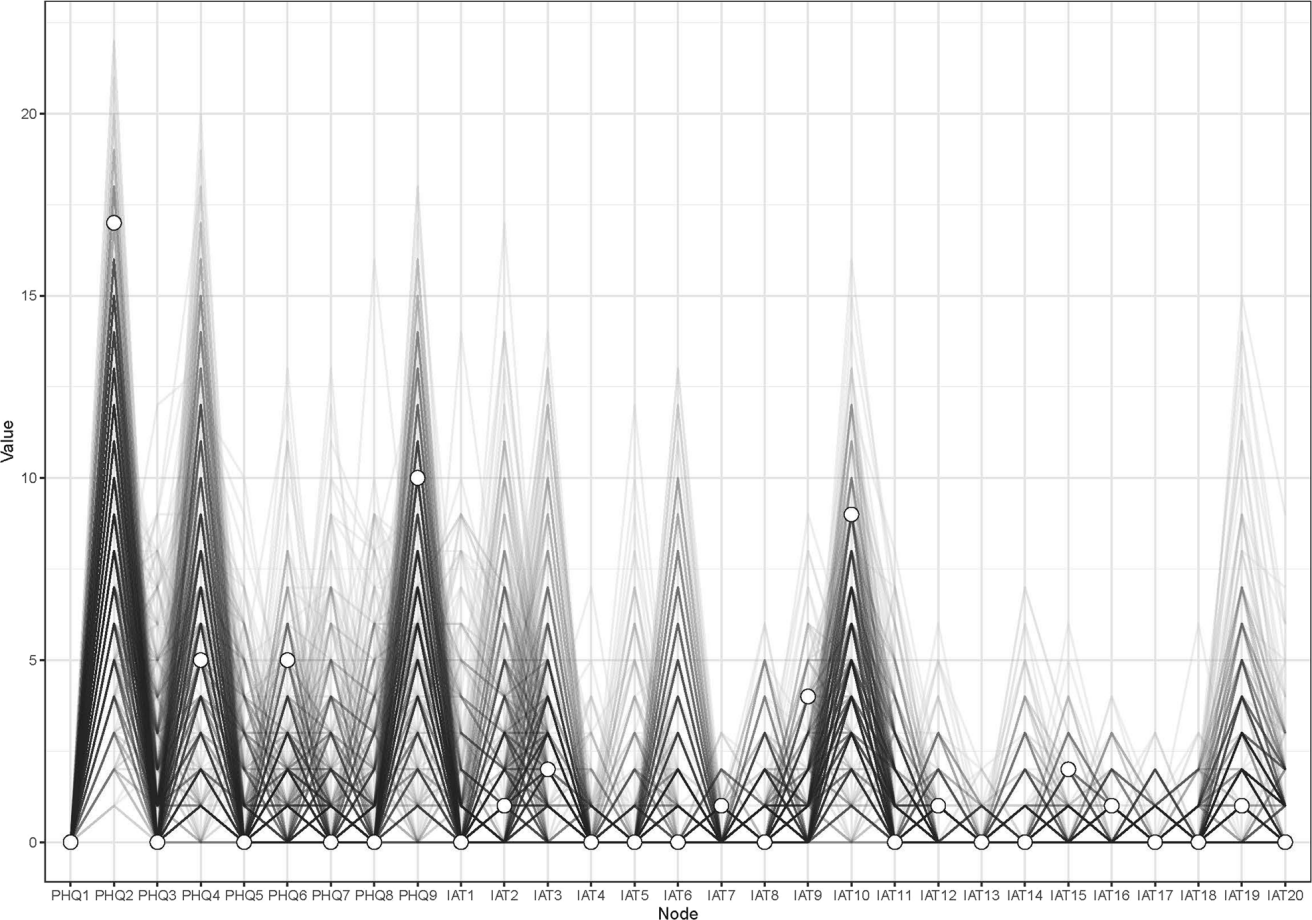
Node-specific predictive betweenness.

Previous studies found that “Anhedonia” was strongly associated with the development of IA behaviors in both adults and adolescents [7, 66, 67]. The bootstrapping procedure showed that the estimation of node-specific predictive betweenness (i.e., items that more often lie on the shortest pathways from “Anhedonia” (PHQ1) to other nodes) was considerably less precise than that of other features of the network. Figure 4 shows the node-specific predictive betweenness values for each node in the network. The white dots represent the node-specific predictive betweenness in the study sample, while the black lines represent the variability of the measure across 1000 nonparametric bootstrap iterations. PHQ2 (“Sad Mood”) had the highest node-specific predictive betweenness score (Fig. 4 and Supplementary Fig. S2).

### Network comparison tests by gender

We conducted a gender comparison analysis between genders since previous studies found gender differences in the relationship between IA and residual depressive symptoms among adolescents [68]. The comparison of network models by gender did not find significant differences in the network global strength (network strength: 15.02 in female participants; 13.82 male participants; 13.69, *P* = 0.521) and edge weights (M = 0.16, *P* = 0.331, Supplementary Figs. S3, S4).

### The confounding effects of age and gender

A previous meta-analysis indicated that gender and age could moderate IA in adolescents [69]. Hence, following previous studies [70, 71], the IA and depression symptoms network model and structure indexes were re-estimated. As compared with the original network, there was no significant structural change after controlling for age and gender (strength: r_s_ = 0.03[−0.33-0.40]) (supplementary Fig. S5).

## Discussion

This was the first study that investigated comorbid IA and RDS in a large sample of clinically stable adolescents with psychiatric disorders using network analysis. Overall, comorbid IA was common in this sample during the COVID-19 pandemic. The nodes IAT15 (“Preoccupation with the Internet”), PHQ2 (“Sad mood”), and PHQ1 (“Anhedonia”) were the most central symptoms in the network model of comorbid IA and RDS. Bridge symptoms included IAT10 (“Sooth disturbing about your Internet use”), PHQ9 (“Suicide ideation”), and IAT3 (“Prefer the excitement online to the time with others”). Additionally, PHQ2 (“Sad mood”) was the main node linking Anhedonia to the IA community.

Internet addiction is usually associated with a preoccupation with the Internet; i.e., adolescents with IA often think about using the Internet when they are offline and often fantasize about surfing on the Internet even if they are concentrating on other things [72]. Not surprisingly, adolescent psychiatric patients with comorbid IA have crucial impairment in their ability to control their Internet use. This network analysis identified the node “Preoccupation with the Internet” as the most central symptom, which supports the proposed diagnostic criteria for IA in adolescents [73] as well as the central aspect of the compensatory Internet use (CIU) model [74]. Our findings are also consistent with the network analysis of adolescents with problematic smartphone use [40, 41]. Due to the strict public health measures (e.g., lockdowns and closing schools) during the COVID-19 pandemic, adolescents often had online schooling, reduced outdoor physical exercises, and increased social distancing, all of which could lead to increased time using the Internet [10, 75]. Further, adolescents with psychiatric disorders usually suffer from cognitive dysfunction, such as difficulty in inhibitory control over Internet use [76, 77]. Previous studies in adolescent psychiatric patients found that excessive use of the Internet might be associated with abnormal changes in brain regions and systems, including the prefrontal cortex and limbic system, which are involved in behavioral and emotional control [78, 79].

“Sad mood” and “Anhedonia” were also central symptoms in the IA-RDS network model, which is consistent with previous findings [80, 81] that anhedonia and sad mood (i.e., diminished pleasure in normally enjoyable activities) were linked to the etiology of IA among adolescents. Additionally, the mental health impact of the COVID-19 pandemic related to the suspension of classroom teaching, daily lifestyle changes, and stress on the economic and health systems could increase the likelihood of sad mood among adolescents [82, 83], which in turn might strengthen the associations of certain depressive symptoms such as sad mood with IA.

The common mechanism involving dopamine in both depression and IA could presumably explain the association of IA with anhedonia and sad mood. For instance, the occurrence of depressive symptoms was associated with decreased levels of dopamine secretion [84]. Levels of D2 dopamine receptors in the caudate nucleus and putamen (the two main parts of the striatum) were reduced in many patients with IA [85]. Similarly, compared with controls, the dopamine transporter (DAT) expression level and dopamine uptake rate in the striatum of IA people were found to be significantly reduced in this population [86].

The bridge symptoms in this network model included “Sooth disturbing about your Internet use”, “Suicide ideation”, and “Preferring the excitement online to the time with others”. A recent network analysis of problematic smartphone use in adolescents revealed that bridge symptoms included “Peer attitudes towards smartphone use”, “Peer pressure for smartphone use”, and “Fear of missing out” [41], which are not consistent with our findings probably due to differences in study samples (clinically stable psychiatric adolescents vs. healthy adolescents). The IAT node “Sooth disturbing about your Internet use” included in the “Reliance on online life” domain of the IAT indicates an increase in addiction. The comorbidity of addiction behaviors (e.g., Internet addiction) and psychiatric disorders (e.g., depression) are common [14]. Previous studies found that heavy Internet use was utilized by patients to alleviate psychiatric symptoms [14].

Suicidal ideation is one of the key symptoms of depressed mood [87]. The social, economic, and health impacts of the COVID-19 pandemic, which greatly affected the daily routine of young people and their families, were associated with increased risk of suicidality in patients with psychiatric disorders such as MDD [88, 89], BD and schizophrenia patients [90], and even in adolescents without psychiatric problems [91, 92]. Given the easy access to the Internet, adolescents with IA could obtain online information about suicide easily, and they may further discuss suicide issues with others [93, 94]. Some studies found that anonymous online communications may facilitate the dissemination of suicide information without being criticized or judged [95]. Furthermore, adolescents with IA are likely to join online groups with individuals having similar interest in suicide, leading them to express suicidal thoughts and behaviors, thereby gaining group recognition and acceptance [93, 95]. The node “Preferring the excitement online to the time with others” included in the “Relationships domain” in the IAT refers to preferring online relationships with decreasing self-control. Self-isolation due to social withdrawal and impaired control and decision-making could increase the risk of addictive behaviors in adolescents with psychiatric disorders [14]. Furthermore, addiction behaviors could further worsen the RDS symptoms in adolescents with psychiatric disorders [14].

Previous studies [7, 66, 67] on comorbid psychiatric syndromes found that “Anhedonia” was commonly reported to link between different symptom communities as a key node. As a depressive symptom and also a trait vulnerability in depression [96], anhedonia is associated with a lowered sensitivity to reward [97, 98] and reduced activation in the ventral striatum in response to pleasant or rewarding stimuli [81, 99], which are similarly found in those with IA [80, 100]. Anhedonia was associated with lower sensitivity to rewards [97], while IA was also associated with lower reward sensitivity [80, 100], both of which could explain the close association between anhedonia and IA [67]. We found the closest pathway between anhedonia and IA was through sad mood, which suggests that “Sad mood” may be a main connector between PHQ1 (“Anhedonia”) and the IA community. A sad mood is not only a symptom of depression but is also conceptualized as a trait vulnerability to depression; therefore, the trait of a sad mood may increase the susceptibility to IA [101]. Patients with RDS may tend to use the Internet compulsively, which provides a source of reward at minimal cost as a means to offset deficits in hedonic experience [100].

The results of the network analysis on RDS and IA have important implications. Regular screening for RDS and IA among adolescents with psychiatric patients is important even when they are clinically stable. Effective interventions that target the identified central and bridge symptoms should be provided to those with comorbid RDS and IA to reduce the risk of negative outcomes such as impaired functioning and suicidality. For example, cognitive-behavioral therapy (CBT) approaches that such as writing a diary of Internet activity and general time management skills (i.e., focusing on offline realities, and controlling or reducing the time spent online [102]), behavioral activation and restructuring cognitive distortions (i.e., strengthening control processes and changing biases in attention and action tendencies [103]) may be effective in reducing the risk of IA [104, 105]. Other effective measures may include online or face-to-face family-based interventions that can enhance peer-to-peer offline communication, improve family functions relationships, and educate families on monitoring Internet use [106]. In addition, reality therapy (e.g., targeting goal-directed choices and self-control by helping individuals reflect on their behaviors, evaluating their choices, and planning to choose effective options) may help individuals control their impulsivity and other behaviors related to Internet use [107].

The strengths of this study included the large sample size, multicenter study design, and use of network analysis with reliable findings. However, certain limitations should also be acknowledged. First, a cross-sectional design was used; therefore, causal relationships between comorbid RDS and IA could not be inferred. Second, this study focused on clinically stable psychiatric adolescents, which limits the generalizability of findings to other patients in different phases of psychiatric disorders. Third, for logistical reasons, certain factors associated with comorbid IA, such as comorbidities, and use of psychotropic medications, were not recorded. Finally, the type and doses of psychotropic medications used were also not recorded.

## Conclusion

In conclusion, the core symptoms (“Preoccupation with the Internet”, “Sad mood”, and “Anhedonia”) and bridge symptoms (“Sooth disturbing about your Internet use”, “Suicide ideation”, and “Prefer the excitement online to the time with others”) identified in this study could be targets for prevention and treatment of comorbid IA and RDS in clinically stable adolescents with major psychiatric disorders during the COVID-19 pandemic.

## Supplementary information

supplementary materials
