# Supplementary material for: Internet addiction and residual depressive symptoms among clinically stable adolescents with major psychiatric disorders during the COVID-19 pandemic: a network analysis perspective
Source: Transl Psychiatry. 2023 Jun 3;13:186. doi: 10.1038/s41398-023-02468-5 (PMC10238780; doi:10.1038/s41398-023-02468-5)

**Supplementary Material**

Figure S1. Nonparametric bootstrapped difference test

Figure S2. Average case-drop bootstraps of node-specific betweenness, based on 1000 iterations

Figure S3. Estimated network model for the Internet addiction and residual depressive symptoms in females and males

Figure S4. Comparison of network properties between females and males

Figure S5. Visual representation of the network after controlling for age and gender.

Table S1. English version of Internet addiction test

|  | Never | Seldom | Occasionally | Frequently | Very often | Always |
| --- | --- | --- | --- | --- | --- | --- |
| 1.How often do you find that you stay online longer than you intended? | 0 | 1 | 2 | 3 | 4 | 5 |
| 2.How often do you neglect household chores to spend more time online? | 0 | 1 | 2 | 3 | 4 | 5 |
| 3.How often do you prefer the excitement of the internet to intimacy with your partner? | 0 | 1 | 2 | 3 | 4 | 5 |
| 4.How often do you form new relationships with fellow online users? | 0 | 1 | 2 | 3 | 4 | 5 |
| 5.How often do others in your life complain to you about the amount of time you spend online? | 0 | 1 | 2 | 3 | 4 | 5 |
| 6.How often do your grades or school work suffer because of the amount of time you spend online? | 0 | 1 | 2 | 3 | 4 | 5 |
| 7.How often do you check your email before something else that you need to do? | 0 | 1 | 2 | 3 | 4 | 5 |
| 8.How often does your job performance or productivity suffer because of the internet? | 0 | 1 | 2 | 3 | 4 | 5 |
| 9.How often do you become defensive or secretive when anyone asks you what you do online? | 0 | 1 | 2 | 3 | 4 | 5 |
| 10.How often do you block out disturbing thoughts about your life with soothing thoughts of the internet? | 0 | 1 | 2 | 3 | 4 | 5 |
| 11.How often do you find yourself anticipating when you will go online again? | 0 | 1 | 2 | 3 | 4 | 5 |
| 12.How often do you fear that life without the internet would be boring, empty, and joyless? | 0 | 1 | 2 | 3 | 4 | 5 |
| 13.How often do you snap, yell, or act annoyed if someone bothers you while you are online? | 0 | 1 | 2 | 3 | 4 | 5 |
| 14.How often do you lose sleep due to being online? | 0 | 1 | 2 | 3 | 4 | 5 |
| 15.How often do you feel preoccupied with the internet when off-line, or fantasize about being online? | 0 | 1 | 2 | 3 | 4 | 5 |
| 16.How often do you find yourself saying “just a few more minutes” when online? | 0 | 1 | 2 | 3 | 4 | 5 |
| 17.How often do you try to cut down the amount of time you spend online and fail? | 0 | 1 | 2 | 3 | 4 | 5 |
| 18.How often do you try to hide how long you’ve been online? | 0 | 1 | 2 | 3 | 4 | 5 |
| 19.How often do you choose to spend more time online over going out with others? | 0 | 1 | 2 | 3 | 4 | 5 |
| 20.How often do you feel depressed, moody, or nervous when you are off-line, which goes away once you are back online? | 0 | 1 | 2 | 3 | 4 | 5 |

Table S2. English version of 9-items Patient Health Questionnaire

|  | Not at all | Several days | More than half the days | Nearly every day |
| --- | --- | --- | --- | --- |
| 1.Little interest or pleasure in doing things? | 0 | 1 | 2 | 3 |
| 2.Feeling down, depressed, or hopeless? | 0 | 1 | 2 | 3 |
| 3.Trouble falling or staying asleep, or sleeping too much? | 0 | 1 | 2 | 3 |
| 4.Feeling tired or having little energy? | 0 | 1 | 2 | 3 |
| 5.Poor appetite or overeating? | 0 | 1 | 2 | 3 |
| 6.Feeling bad about yourself-or that you are a failure or have let yourself or your family down? | 0 | 1 | 2 | 3 |
| 7.Trouble concentrating on things, such as reading the newspaper or watching television? | 0 | 1 | 2 | 3 |
| 8.Moving or speaking so slowly that other people could have noticed? Or so fidgety or restless that you have been moving a lot more than usual? | 0 | 1 | 2 | 3 |
| 9.Thoughts that you would be better off dead, or thoughts of hurting yourself in some way? | 0 | 1 | 2 | 3 |

Table S3. Means, standard deviations, skewness, and kurtosis.

| Item | M | SD | Skewness | kurtosis |
| --- | --- | --- | --- | --- |
| PHQ1 | 2.09 | 1.15 | 0.56 | -1.18 |
| PHQ2 | 1.99 | 1.10 | 0.72 | -0.88 |
| PHQ3 | 2.02 | 1.14 | 0.68 | -1.01 |
| PHQ4 | 2.09 | 1.12 | 0.57 | -1.09 |
| PHQ5 | 1.89 | 1.10 | 0.87 | -0.70 |
| PHQ6 | 2.02 | 1.17 | 0.66 | -1.12 |
| PHQ7 | 1.90 | 1.09 | 0.86 | -0.67 |
| PHQ8 | 1.77 | 1.05 | 1.07 | -0.26 |
| PHQ9 | 1.74 | 1.07 | 1.15 | -0.15 |
| IAT-1 | 2.65 | 1.43 | 0.30 | -1.30 |
| IAT-2 | 2.27 | 1.29 | 0.68 | -0.69 |
| IAT-3 | 2.28 | 1.42 | 0.73 | -0.87 |
| IAT-4 | 1.68 | 1.06 | 1.61 | 1.77 |
| IAT-5 | 2.41 | 1.42 | 0.56 | -1.06 |
| IAT-6 | 2.10 | 1.28 | 0.90 | -0.35 |
| IAT-7 | 1.78 | 1.11 | 1.39 | 1.05 |
| IAT-8 | 1.82 | 1.12 | 1.24 | 0.63 |
| IAT-9 | 2.11 | 1.34 | 0.92 | -0.45 |
| IAT-10 | 2.35 | 1.42 | 0.62 | -1.01 |
| IAT-11 | 2.03 | 1.29 | 1.00 | -0.25 |
| IAT-12 | 1.23 | 1.36 | 0.77 | -0.71 |
| IAT-13 | 1.99 | 1.23 | 1.01 | -0.12 |
| IAT-14 | 1.99 | 1.26 | 1.09 | 0.03 |
| IAT-15 | 2.01 | 1.18 | 1.03 | 0.10 |
| IAT-16 | 2.14 | 1.27 | 0.86 | -0.42 |
| IAT-17 | 1.88 | 1.18 | 1.17 | 0.30 |
| IAT-18 | 1.72 | 1.11 | 1.45 | 1.12 |
| IAT-19 | 1.99 | 1.34 | 1.09 | -0.16 |
| IAT-20 | 1.76 | 1.15 | 1.42 | 0.92 |

Figure S1. Nonparametric bootstrapped difference test

A:


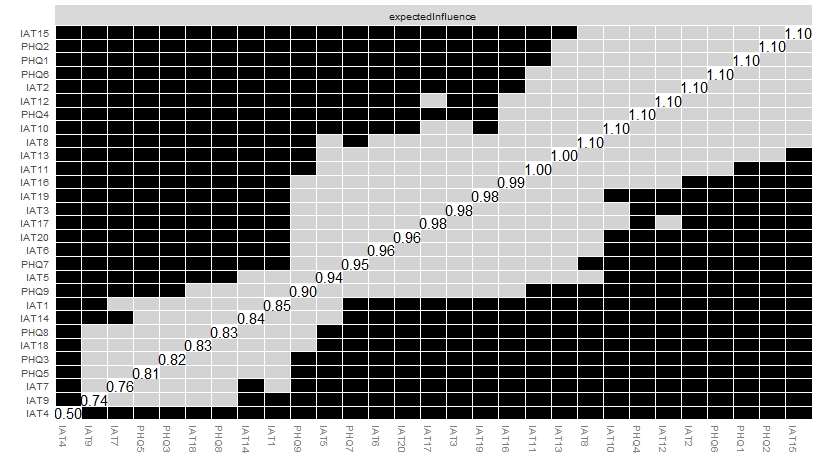


B:


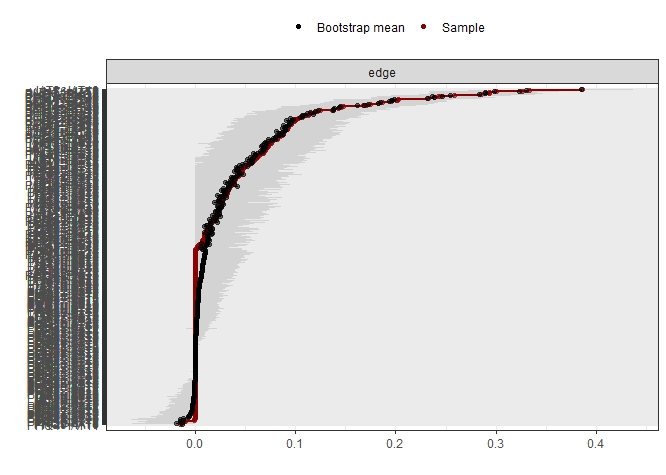


Bootstrapped difference tests between edge weights in the network. Gray boxes indicate edges that do not significantly differ from one another. Black boxes represent edges with a significant difference from one another.

Figure S2. Average case-drop bootstraps of node-specific betweenness, based on 1000 iterations


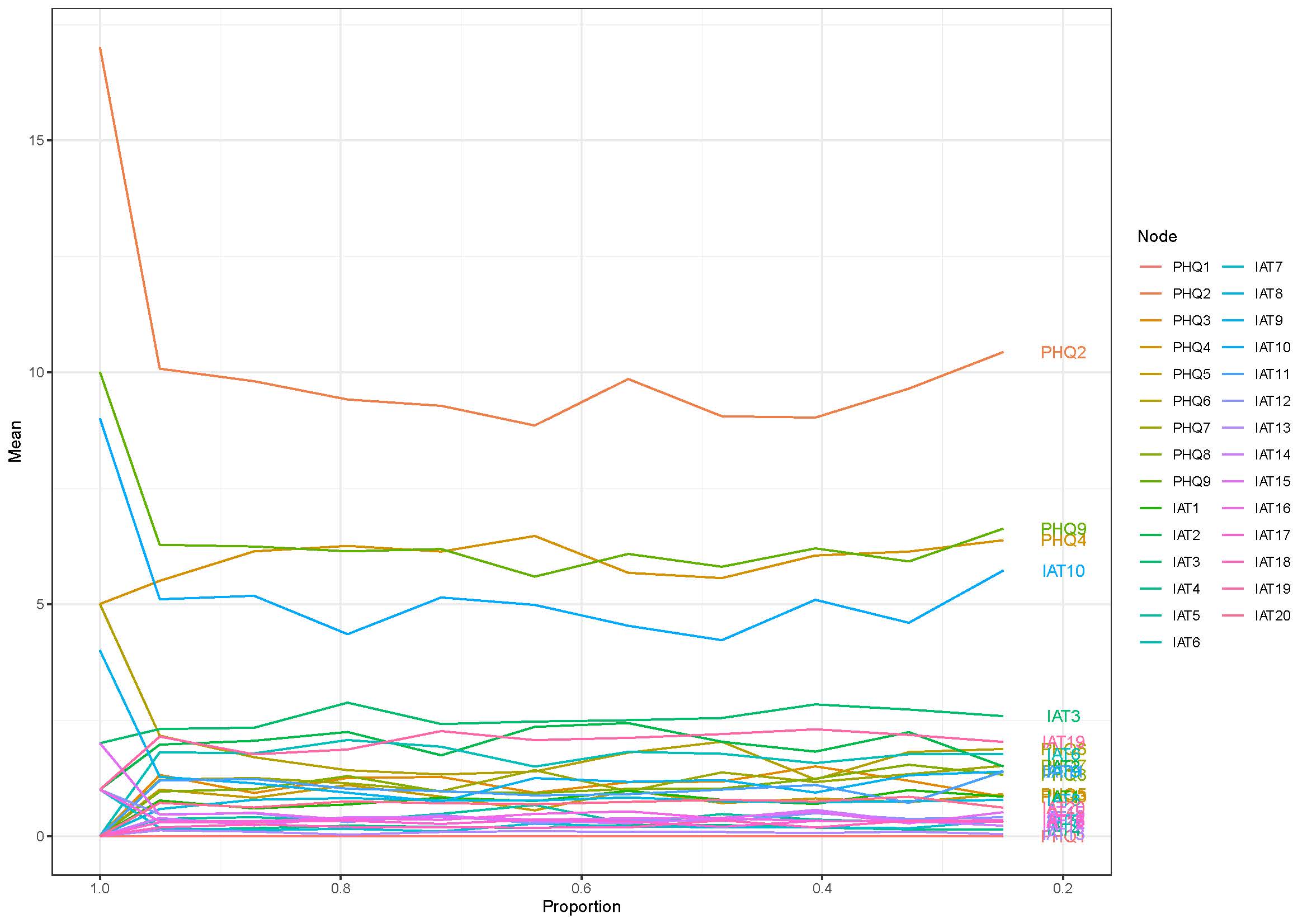


The lines represent how node-specific betweenness (i.e., how often a node lies on the pathways between two other nodes, of which one is always Suicide ideation) changes for each variable when dropping different proportions of the data.

Figure S3. Estimated network model for the Internet addiction and residual depressive symptoms in females and males


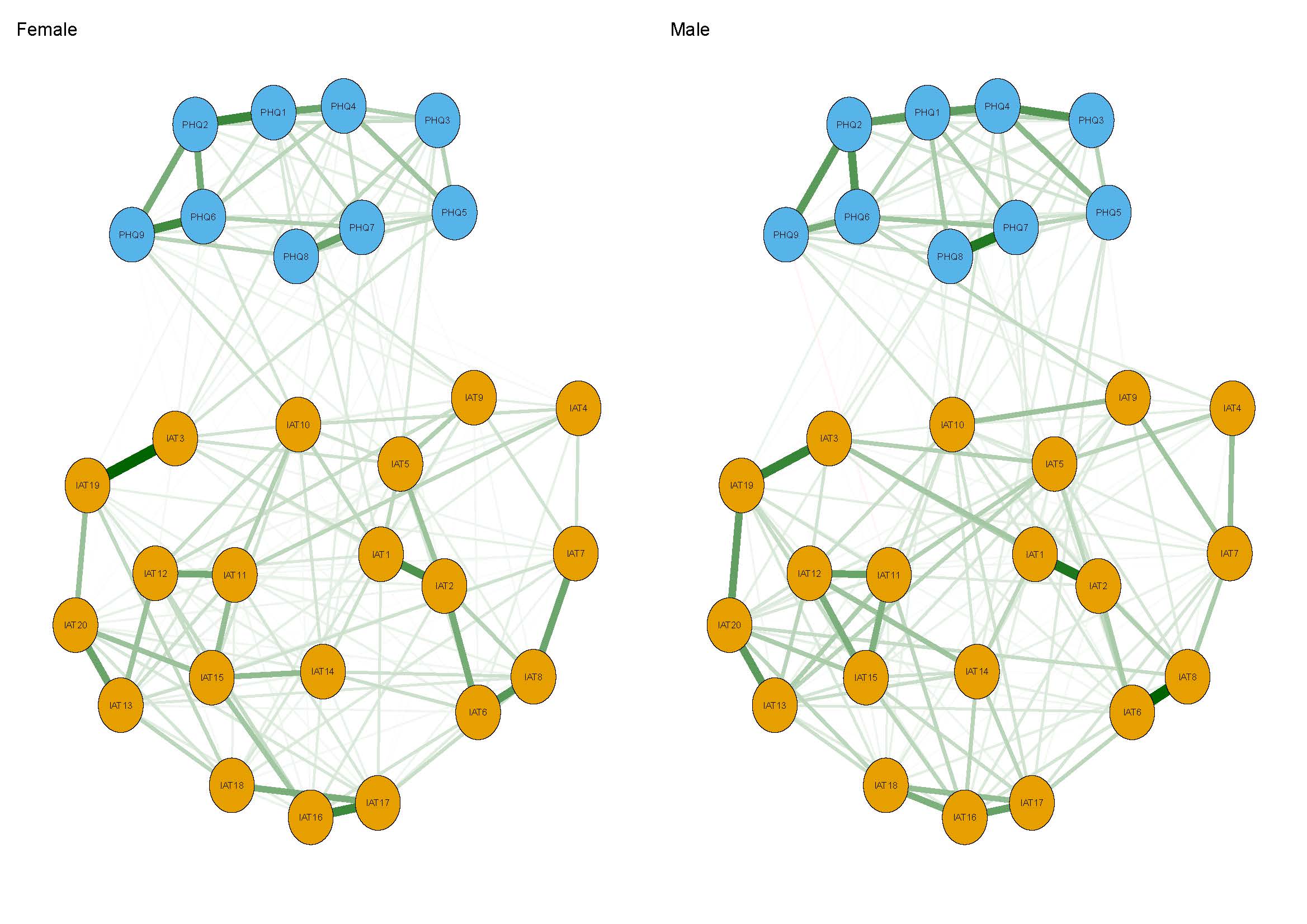


Figure S4. Comparison of network properties between females and males


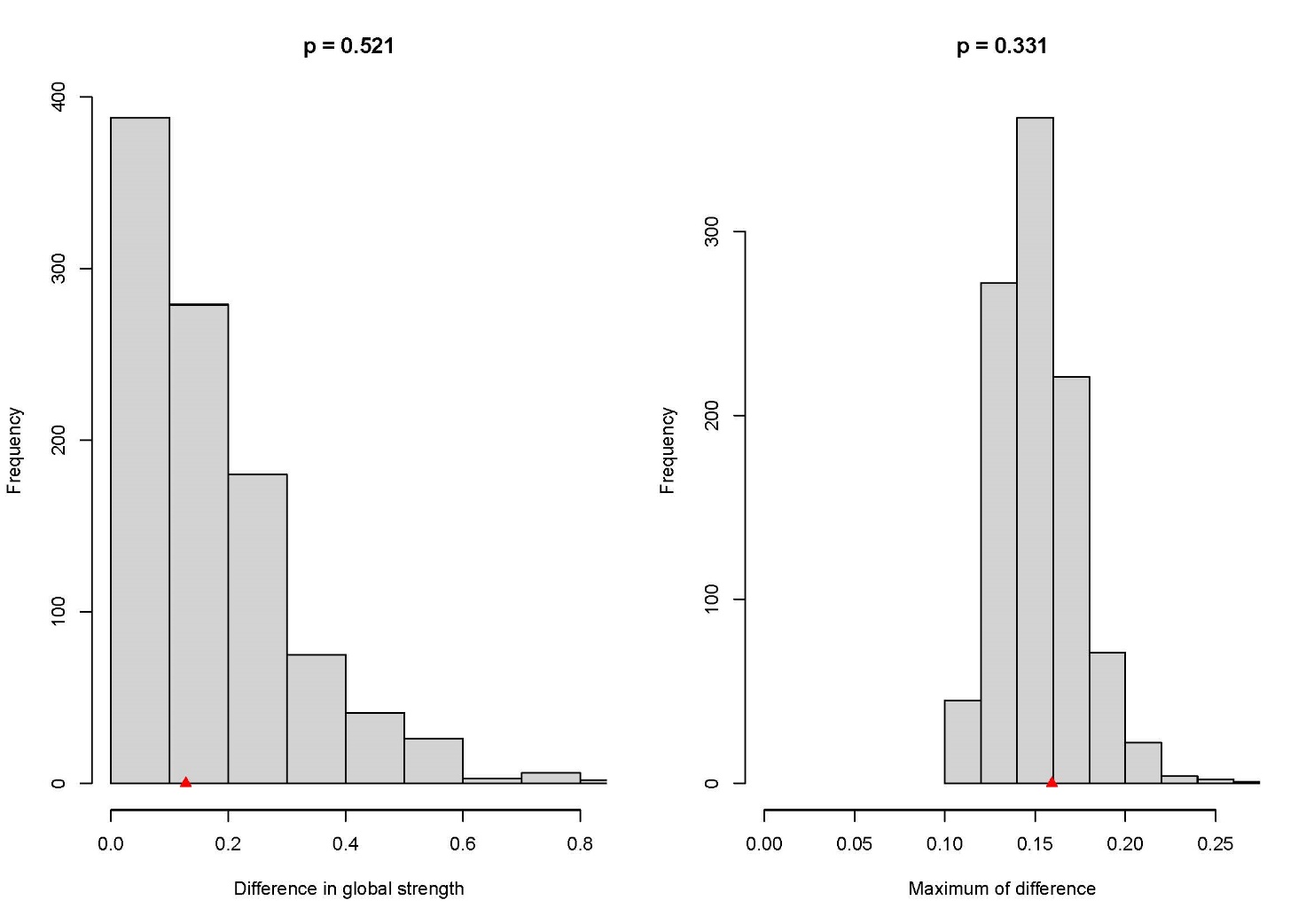


Figure S5. Visual representation of the network after controlling for age and gender.


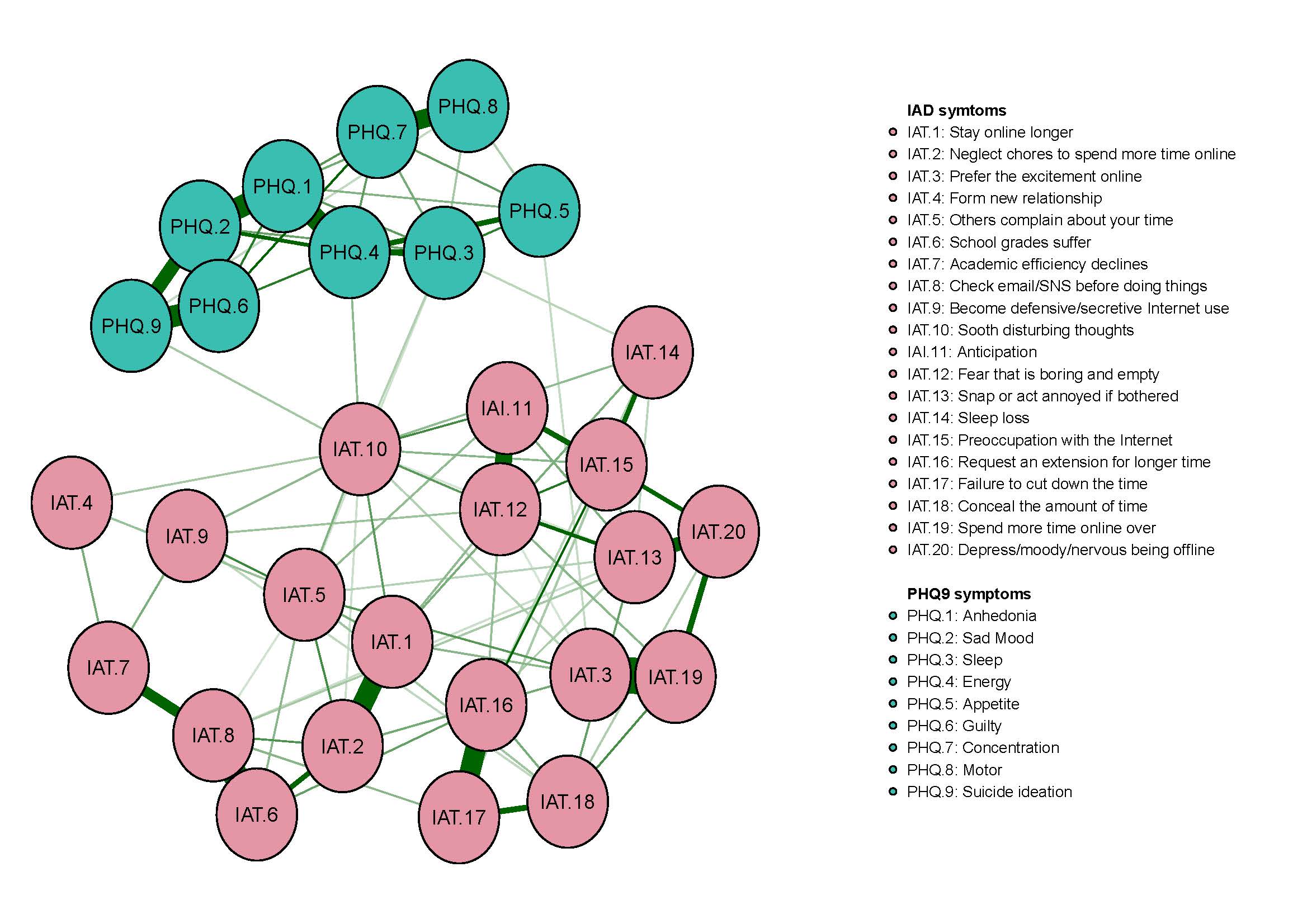

Supplement: Supplementary file 1 — supplementary materials [file 41398_2023_2468_MOESM1_ESM.docx]
